# Effect of Probiotics Supplementations on Health Status of Athletes

**DOI:** 10.3390/ijerph16224469

**Published:** 2019-11-13

**Authors:** Bhagavathi Sundaram Sivamaruthi, Periyanaina Kesika, Chaiyavat Chaiyasut

**Affiliations:** Innovation Center for Holistic Health, Nutraceuticals, and Cosmeceuticals, Faculty of Pharmacy, Chiang Mai University, Chiang Mai 50200, Thailand; p.kesika@gmail.com

**Keywords:** probiotics, athletes, upper respiratory tract infections, intestinal permeability, gastrointestinal symptoms

## Abstract

Athletes are prone to several health complications, including upper respiratory tract infections, allergies, and gastrointestinal discomforts during practice and after the performance due to the intense exercise, travel, insufficient rest and restricted food consumption. Probiotics are well known as complementary therapeutic and health supplements for several diseases and disorders. Studies suggest that the intervention of probiotics improved the health status of elite athletes, but the results are not consistent in all the studies. The beneficial effect of probiotic supplementation profoundly relies on species or strain, dose, duration, form, and host physiology. The manuscript summarizes the effect of probiotic supplementation on health status of athletes. The literature was collected from PubMed, Scopus, Web of Science, and Google Scholar using the search term “probiotic and athletes”. As per the literature survey, probiotic supplementation improved the intestinal permeability, immune system, intestinal microbiota, inflammatory system, reduced the severity and incidence of respiratory tract infections, and duration of gastrointestinal symptoms. Several studies were conducted on *Lactobacillus* species and the outcomes were found to be species- or strain-specific. More studies are required to know the detailed mechanism behind the beneficial effect of probiotic intervention in athletes. Further studies are desired on formulation and optimization of probiotic supplements to develop generalized and personalized sports supplements to boost the overall health and enactment of elite athletes.

## 1. Introduction

Intense training, excessive exercise, insufficient rest, travel and improper nutrition are the factors that induce stress in athletes and make them prone to several health complications like immune depression, inflammatory dysregulation, increased respiratory tract infections, and oxidative and mental stress [1,2,3]. In particular, endotoxemia and gastrointestinal (GI) symptoms were reported in long-distance athletes (marathon, ultra-endurance, and triathlon athletes) [4,5]. In recent years, the researchers are focusing on the health improvement of athletes to prevent and manage the health issues related to their profession with the aid of dietary supplements [6].

Probiotic-based supplements are one such complementary method to improve the athlete’s health. Probiotics are “live microorganisms which, when administered in adequate amounts, confer a health benefit on the host”. Lactic acid bacteria, Bifidobacteria, *Pediococcus*, *Leuconostoc*, *Streptococcus*, *Saccharomyces*, *Bacillus,* and *Enterococcus* are the commonly used probiotic strains [7]. In recent decades, probiotics are recommended as a health supplement to improve health status and manage diseases and disorders [8,9,10,11,12,13,14,15].

Several recent studies have updated the exercise, probiotic and nutritional supplement recommendations for athletes [16,17,18]. The present manuscript narrates the effect of probiotic supplementation on the health status of athletes, possible mechanism of probiotic action, and factors influence the effect of probiotic supplementation. The scientific reports were collected from PubMed, Scopus, Web of Science, and Google Scholar using the search term “probiotic and athletes”. The scientific reports that are available with details of probiotic dose and are relevant to the scope were selected and included in the review preparation (number of articles reviewed =19). These include the effect of probiotic supplements on the health status of athletes in terms of reducing exercise induced stress; improving the host immunity; reducing the symptoms of GI and upper respiratory tract infections; and/or improving intestinal microbiota and intestinal permeability.

## 2. Effect of Probiotic Supplementation on the Health Status of Athletes

### 2.1. Single-Strain Probiotic Intervention

Extensive exercise may influence the athlete health due to exercise-induced stress, which causes gastrointestinal (GI) symptoms and is also associated with increased risk of upper respiratory tract infections (URTI). Marathon runners might suffer from respiratory tract infections during the training period and after the marathon race. Kekkonen et al. [19] studied the effect of *Lactobacillus rhamnosus* GG (Gorbach-Goldin) on the GI symptoms and URTIs in marathon runners during the training period. Marathon runners were supplemented with *L. rhamnosus* GG (4 × 10^10^ CFU per day in the form of milk-based fruit drink or 10^10^ CFU per day in the form of capsule) for three months during the training period. After the race, the subjects were followed up for two weeks to assess the changes in their healthy days, and days with GI-symptoms and URTIs. There was a slight increase in the number of healthy days in the probiotic group compared to that of the placebo group (*p* = 0.82). The probiotic group and placebo group showed no significant difference in the number of GI symptoms and respiratory infections. However, the probiotic-supplemented group showed a 33% reduction (during the training period) and 57% reduction (during the follow-up period) in duration of GI-symptom episodes when compared to the placebo group. There were no changes observed in hematological parameters such as hemoglobin, erythrocytes, hematocrit during the training and follow-up periods in both the probiotics and placebo groups. The results suggested that *L. rhamnosus* GG supplementation showed no beneficial effect on the prevalence of GI-symptom episodes and URTI, but reduced the duration of GI-symptom episodes in healthy marathon runners [19].

Clancy et al. [20] investigated the effect of *L. acidophilus* LAFTI^®^L10 in the fatigued athletes, who were diagnosed with a low level of interferon-γ (IFN-γ) secretion and increased risk of viral (Epstein–Barr virus) infection. Supplementation of *L. acidophilus* LAFTI^®^L10 (2 × 10^10^ CFU per day) for four weeks increased the secretion of IFN-γ from whole blood cells in fatigued athletes comparatively to the level of healthy subjects. Probiotic intervention also increased salivary IFN-γ level in both healthy and fatigued athletes. However, the levels of salivary immunoglobulin A (IgA), and secretory interleukins (IL-4, IL-12) were not altered significantly after probiotic intervention in both healthy and fatigued athletes. The study showed the beneficial effect of probiotic therapy by revealing the reversal of IFN-γ reduction in fatigued athletes and enhancement of mucosal IFN-γ level in healthy athletes upon supplementation of *L. acidophilus* [20].

Gleeson et al. [21] investigated the effect of *L. casei* Shirota on the incidence of URTI in endurance athletes. The supplementation of fermented milk containing *L. casei* Shirota (1.3 × 10^10^ CFU per day) for sixteen weeks significantly reduced the incidence of URTI, while no changes in severity of URTI was observed among the probiotic and placebo subjects. The level of IgA was found to increase during probiotic supplementation, whereas the changes in immunoglobulin G (IgG), immunoglobulin M (IgM), and total immunoglobulin were not correlated with treatment. The changes in cytokines (IL-2, IL-4, IL-6, IL-8, IL-1β, and IFN-γ) levels and blood cell counts were statistically not associated with treatment. The results suggested that the consumption of *L. casei* Shirota reduced the incidence of URTI in athletes during the winter training period [21]. Subsequently, Gleeson et al. [22] reported that the supplementation of fermented milk containing *L. casei* Shirota (1.3 × 10^10^ CFU per day) for twenty weeks did not significantly reduce the episodes of URTI in athletes. However, Epstein–Barr virus and cytomegalovirus antibody titers were reduced in the probiotic supplemented group. Regular consumption of *L. casei* Shirota could improve the immune status of endurance athletes [22]

Michalickova et al. [23] examined the effect of *L. helveticus* Lafti L10 on the mucosal and humoral immunity in elite athletes. Supplementation of probiotic strain *L. helveticus* Lafti L10 (2 × 10^10^ CFU per day) for fourteen weeks significantly improved the immune status of elite athletes. Total IgM levels increased significantly, and total IgG levels were found to be unaltered in both groups. Antibacterial-specific IgM levels was found to be unaltered. Anti-*Enterococcus faecalis* IgG levels were found to be reduced significantly in the placebo group compared to that of the probiotic group. The results revealed that *L. helveticus* Lafti L10 supplementation might control the URTI by improving the host immune system [23].

West et al. [24] investigated the effect of *L. fermentum* VRI-003 PCC^®^ (Probiomics Ltd, Sydney, Australia) on the symptoms of GI and URTIs in athletes (healthy, well-trained individuals such as cyclists). Competitive cyclists were supplemented with *L. fermentum* (PCC^®^) (1 × 10^9^ CFU per day) for eleven weeks, and the changes in the severity of illness, fecal *Lactobacillus* count, and serum cytokines were measured. The training period, duration and intensity of exercise, workload, and dietary intake were not significantly differed among the subjects. The total *Lactobacillus* load was increased in the probiotic group, especially in man subjects while no difference was observed in *Bifidobacterium*, *Bacteroides fragilis, Escherichia coli*, and *Clostridium coccoides* load. The pro-inflammatory (Il-8, tumor necrosis factor alpha (TNF-α), IFN-γ, granulocyte macrophage-colony stimulating factor), anti-inflammatory (IL-1ra, IL-10), and immune-regulatory (IL-6) cytokines level were not significantly altered during treatment, but the possible reduction was observed in all tested cytokines when compared to placebo. The mean difference of cytokines was found to be reduced in female subjects than male volunteers. *L. fermentum* supplementation reduces the URTI symptoms in terms of severity, number of episodes, severity-days in male subjects. The severity of the GI symptoms was found to be reduced in male athletes during higher training loads upon probiotic treatment. The link between illness severity and changes in fecal microbiota has not been explained. The results suggest that *L. fermentum* (PCC^®^) supplementation possibly improved the health status (GI-symptoms, and respiratory illness) of male competitive cyclists when compared to female counterparts, possibly by reducing the exercise-induced immune disquiets [24].

The influence of supplementation of *L. fermentum* VRI-003 on respiratory health and immune system of healthy elite male distance runners was studied by Cox et al. [25]. The subjects were supplemented with either 1.2 × 10^10^ CFU per day of probiotics or placebo capsules for 28 days (1st treatment month). After 28 days of washout period, subjects of probiotic, and placebo groups received the placebo capsules, and *L. fermentum* for next 28 days (2nd treatment month) i.e., those who had probiotic supplementation in the 1st treatment were supplemented with placebo capsules for 2nd treatment, and vice versa. After 28 days of the 2nd treatment, the subjects were followed-up for two weeks. The number of days and severity of URTI symptoms were reduced during probiotic supplementation. Changes in the level of IgA, IgA1, IL-4, and IL-12 in the probiotic group were found to be not significantly different than that of the placebo group, whereas a significant level of increase in IFN-γ was observed in the *L. fermentum* VRI-003-supplemented group when compared to the placebo group. The results suggested that the reduction of incidence and severity of URTI symptoms in the probiotic group was correlated with an elevated level of IFN-γ [25], as reported earlier by Clancy et al. [20].

Komano et al. [26] investigated the effect of heat-killed *Lactococcus lactis* JCM 5805 supplementation on the URTIs and fatigue in athletes. The supplementation of heat-killed *L. lactis* JCM 5805 strain (10 × 10^10^ CFU per day) for thirteen days significantly reduced the incidence and symptoms (sneeze or running nose) of URTI in active athletes via plasmacytoid dendritic cells activation. The muscle damage markers (creatine phosphor kinase and lactate dehydrogenase) and stress markers (adrenaline and salivary cortisol) were increased after exercise, but not influenced by any of the intervention. The performance of the athletes was not affected by probiotic or placebo interventions. The results suggested that the supplementation of heat-killed *L. lactis* JCM 5805 could reduce the incidence of URTI and control the fatigue in athletes during high-intensity exercise [26].

Huang et al. [27] studied the effect of *L. plantarum* PS128 on the health status of triathletes. Triathletes were supplemented with *L. plantarum* PS128 during the training period and the effect of probiotic supplementation on inflammation, oxidative stress and performance of the athletes were assessed. The exercise-induced elevation of TNF-α, IL-6, and IL-8 were suppressed in the probiotic group, and the effect was improved after the resting period. The probiotic supplementation increased (during training period) and reduced (during the resting period) the level of thioredoxin, component 5a, myeloperoxidase. Improved exercise performance was observed in the probiotic-supplemented group as compared to the placebo. The plasma-branched amino acid content was increased after probiotic intervention in athletes. Overall, *L. plantarum* PS128 supplementation improved the performance, oxidative stress and inflammatory system of triathletes [27].

### 2.2. Multispecies Probiotic Intervention

Martarelli et al. [28] investigated the effect of probiotic supplementation on the oxidative stress in athletes. The antioxidant potential of *L. rhamnosus* IMC 501^®^ and *L. paracasei* IMC 502^®^ were confirmed in vitro. Healthy athletes were supplemented with probiotic mixture (*L. rhamnosus* IMC 501^®^ and *L. paracasei* IMC 502^®^; approximately 10^9^ cells per day) for four weeks during an extensive training period. The microbiological investigation showed that the fecal *Lactobacillus* load was significantly increased in the probiotic group compared to baseline and placebo control. Notably, *L. rhamnosus* IMC 501^®^ and *L. paracasei* IMC 502^®^ strains were found in the fecal microbiota of probiotic supplemented group, which was not detected at baseline assessments. The reactive oxygen metabolites level was increased after the exercise period in both groups compared to baseline, but the probiotic supplementation was found to neutralize the reactive oxygen species (ROS). Similarly, the antioxidant potential of the athlete increased during probiotic supplementation. The results suggested that the supplementation *L. rhamnosus* IMC 501^®^ and *L. paracasei* IMC 502^®^ mixture improved the antioxidant status of athletes during the rigorous training period [28].

Coman et al. [29] investigated the effect of symbiotic fermented milk on the health status of athletes. Gym-trained athletes were supplemented with a synbiotic formula containing *L. rhamnosus* IMC 501^®^ and *L. paracasei* IMC 502^®^ (10^9^ CFU per strain; 2 × 10^9^ CFU per day) and oat bran fiber for four weeks. The synbiotic supplementation improved gut health and increased the fecal *Lactobacillus* spp. and *Bifidobacterium* spp. load. The secretory IgA level was increased and lipid oxidation was decreased after the synbiotic intervention when compared to the control group. The study suggested that supplementation of IMC 501^®^, IMC 502^®^, and oat bran fiber improved the gut microbiota, mucosal immunity, and oxidative stress [29].

### 2.3. Multigenus Probiotic Intervention

Pumpa et al. [30] investigated the effect of probiotic supplements Ultrabiotic 60^TM^ (FIT-BioCeuticals Ltd, Australia), and SB (*Saccharomyces boulardi*) Floractiv^TM^ (FIT-BioCeuticals Ltd, Australia) on the severity and incidence of GI and URTIs in elite rugby union players and also examined the association between the host mucosal immunity and stress (salivary biomarkers). Elite rugby union players were supplemented with probiotic formula Ultrabiotic 60^TM^ (*L. rhamnosus*, *L. casei*, *L. acidophilus*, *L. plantarum*, *L. fermentum*, *Bifidobacterium lactis*, *B. bifidum*, *Streptococcus thermophilus*; 6 × 10^10^ CFU in total/capsule) and SB Floractiv^TM^ (*Saccharomyces boulardi*; 250 mg). The total experimental period was 27 weeks; the control period (first stage) includes the first ten weeks (week 1 to week 10). Ultrabiotic 60^TM^ (1 capsule twice a day; 12 × 10^10^ CFU per day) was supplemented during the second stage, including the next seven weeks (week 11 to week 17), and SB Floractiv^TM^ (twice a day) was included in the third stage (week 18 to week 27) of the experimental period. The subjects were examined for baseline values of biomarkers (salivary α-amylase, cortisol, and secretory-IgA) during the first 4 weeks (training camp at week 1 and domestic games at week 2 to week 4) of the control period (1st stage). The probiotic effect and changes in the biomarkers level were examined during the national competition (2nd stage) and international competition (3rd stage). When compared within groups, salivary IgA, and α-amylase level was increased during 2nd stage examination compared to that of the stage 1 examination in both groups (probiotic and placebo), whereas the placebo group showed a reduction in salivary cortisol, IgA, and α -amylase level at the 3rd stage examination when compared with the second stage of examination of the same group. When compared between the groups, the salivary cortisol was observed to be higher in the 1st- and 2nd-stage examination, and α-amylase level was significantly higher in the 3rd-stage examination of the probiotic group compared to that of the placebo group. The incidence of GI and URTIs were reduced during the probiotic supplementation. The results resolved that the consumption of Ultrabiotic 60^TM^ and SB Floractiv^TM^ could improve the immune status of elite rugby players [30].

Salarkia et al. [31] studied the effect of probiotic yogurt on the symptoms of URTI in the female swimmers. The supplementation of probiotic yogurt containing *L. acidophilus, L. delbrueckii* subsp. *bulgaricus, B. bifidum*, and *Streptococcus salivarius* subsp. *thermophilus* (400 mL per day; 4 × 10^10^ CFU per mL) for eight weeks improved the health conditions of female swimmers. Specifically, the duration of infectious symptoms (dyspnea and ear pain) was reduced in the probiotic group compared to the control group supplemented with ordinary yogurt. No significant changes were observed regarding the symptoms such as sore throat, cough, rhinitis, fever, stomachache, vomiting, and diarrhea. The average number of episodes of URTI and digestive disorder were found to be reduced in the probiotic group than that of the control group. The results supported that the consumption of probiotic yogurt improved the respiratory and digestive health of active female swimmers [31].

Haywood et al. [32] examined the effect of probiotic supplement on the URTI and GI episodes in elite rugby male players. The supplementation of probiotic capsule containing *L. gasseri* (2.6 × 10^9^ CFU), *B. bifidum* (0.2 × 10^9^ CFU)*, B. longum* (0.2 × 10^9^ CFU) for four weeks significantly reduced number of self-reported symptoms of URTI and GI episodes in elite rugby players compared to the placebo group, whereas the severity of the symptoms was not improved by probiotic supplementation. Many participants in probiotic groups stated that they did not experience any kind of discomfort during the treatment period. The average number of days of illness was found to be reduced in the probiotic arm compared to the placebo. The study stated that the daily intake of probiotic (*L. gasseri*, *B. bifidum* and *B. longum*) supplement improved the health status of the elite rugby players [32].

Robert et al. [33] studied the effect of multistrain probiotic and prebiotic along with or without antioxidant supplementation on the endotoxin levels and GI permeability in novice long-distance triathletes. A probiotic mixture (30 × 10^9^ CFU per day) containing *L. acidophilus* CUL-60 (10 × 10^9^ CFU), *L. acidophilus* CUL-21 (10 × 10^9^ CFU), *B. bifidum* CUL-20 (9.5 × 10^9^ CFU), *B. animalis* subsp. *lactis* CUL-34 (0.5 × 10^9^ CFU) and prebiotics (fructooligosaccharides; 55.8 mg per day) were supplemented to long-distance triathletes with or without α-lipoic acid (400 mg per day), and N-acetyl-carnitine (600 mg per day) for twelve weeks. The dietary intake was not different among the groups. There were no changes observed in the training load and training strain during the experimental period among the participants. A significant reduction in endotoxin units was observed in the probiotic + prebiotic + antioxidant-supplemented group during the pre-race and post-race measurements, and in the probiotic + prebiotic-supplemented group during post-race measurements. The intestinal permeability was significantly increased in terms of urinary lactulose: mannitol recovery ratio from baseline in the placebo group and insignificantly increased from baseline in both probiotic groups. The GI symptoms were reduced in probiotic groups compared to that of the placebo group, and there was no change in race performances due to the treatments. Collectively, the results supported that the consumption of multistrain probiotic supplements could improve the health status of long-distance runners in terms of GI health. The addition of antioxidants in the supplement may boost the beneficial impacts by reducing the endotoxin levels in athletes during the endurance-training period [33].

Lamprecht et al. [34] investigated the effect of probiotic supplementation on markers of inflammation, oxidation, and intestinal barrier in endurance trained male athletes. Trained athletes (triathletes, runners, cyclists) were supplemented with a probiotic formula (Ecologic^®^ Performance (Winclove B.V., Amsterdam, the Netherlands)) containing *B. bifidum* W23, *B. lactis* W51, *E*. *faecium* W54, *L. acidophilus* W22, *L. brevis* W63, and *L. lactis* W58 (total dose = 10^10^ CFU per day) for fourteen weeks. The influence of probiotic supplementation on intestinal permeability, inflammation and oxidation were assessed at rest and after exercise. Probiotic supplementation reduced the baseline level (the values was found to be slightly above the normal physiological range) of zonulin (biomarker of intestinal permeability) to reach its normal range in the trained athletes. Zonulin level was significantly reduced in the probiotic group when compared to the placebo group. There was no change in total oxidation status, α1-antitrypsin (proteins that protect liver damages), and malondialdehyde levels between the placebo and treatment group irrespective of exercise. The concentration of carbonyl groups on proteins (CP) significantly increased after exercise in both groups; the probiotic supplementation reduced the CP concentration but not at a significant level. The TNF-α level was not significantly affected by either exercise or probiotic supplementation, but a slight reduction was observed in the probiotic group compared to that of the placebo group. The IL-6 level increased after exercise compared to resting values in both probiotic and placebo groups, while treatment did not alter the values significantly. The results collectively suggested that probiotic supplementation could improve the intestinal permeability, reduce the TNF-α level (pro-inflammatory marker), and reduce the level of exercise induced protein oxidation in endurance-trained athletes [34].

Strasser et al. [35] studied the influence of supplementation of the probiotic formula, Ecologic^®^ Performance, on tryptophan-kynurenine metabolism and URTI in trained athletes during the winter season (three months). The probiotic mixture (10^10^ CFU per day) was supplemented for twelve weeks, and the serum level tryptophan, phenylalanine, kynurenine, tyrosine, and neopterin were measured before and after the Ecologic^®^ Performance intervention, as well as during exercise and resting periods. The endurance-training period was significantly high in the probiotic group compared to the placebo. There were no notable changes in food consumption, body composition and anthropometric measurements between probiotic and placebo groups. The exercise-induced tryptophan degradation was reduced after probiotic supplementation, while phenylalanine concentration was not influenced by both exercise and interventions. Kynurenine and neopterin levels were increased after exhausting exercise. The probiotic supplementation showed no significant effect on the neopterin levels, and kynurenine/tryptophan ratio. The rate of incidence of URTI was higher (~2.2 fold) in the placebo group compared to that of the probiotic group. The disease symptoms were correlated with exercise-induced tryptophan degradation, and kynurenine/tryptophan ratio. Together, the results suggested that the probiotic intervention hindered the exercise-induced reduction in tryptophan, and reduced the occurrence of URTI [35] (Table 1).

## 3. Impact of Probiotic Supplementation on Athlete Health: Non-significant Outcomes

Gleeson et al. [36] studied the effect of probiotic supplementation on URTI and mucosal immunity in endurance athletes (runners, cyclists, swimmers, triathletes). Elite athletes were supplemented with *L. salivarius* (2 × 10^10^ CFU per day) for sixteen weeks and there were no significant changes in IgA, lysozyme levels, secretion rates of IgA and lysozyme between the probiotic and placebo groups. Similarly, the count of immune cells (leukocytes, lymphocytes, monocytes, neutrophils) was not found to be notably varied upon probiotic intervention. The incidence, severity, and duration of URTI were not significantly different between probiotic and placebo groups. The results conveyed that the consumption of *L. salivarius* showed no beneficial effect on the host mucosal immunity and the incidence of URTI in elite athletes [36].

Moreira et al. [37] studied the effect of *L. rhamnosus* GG (4 × 10^10^ CFU per day in the form of a milk-based fruit drink or 10^10^ CFU per day in the form of a capsule) supplementation on asthma and allergies associated symptoms in marathon runners during the pollen season (duration of the pollen season is three months, which is prior to the marathon). The results suggested that *L. rhamnosus* GG intervention did not significantly influence the allergic markers (asthma, allergic rhinitis, allergic conjunctivitis, atopic eczema, food allergy, allergen-specific IgE, etc.) during the training period or the marathon race, and the incidence of allergic-related diseases in marathon runners was similar to the general population [37].

## 4. Mechanism of Probiotic Action

Generally, probiotics improved the health status of the consumer by enhancing the intestinal permeability, regulating the immune system, improving the composition of the microbiota, and competitive exclusion of pathogens by reducing the intestinal pH, increasing the short-chain fatty acids and mucus production, and bacteriocin production [2,38].

Intense exercise may cause oxidative stress, muscle damage, inflammation and immune alteration in elite athletes [26,27]. Probiotic supplementation could improve the overall immune status of athletes, and confer several health benefits. Probiotic (*Lactobacillus* species; single-strain [20,25,27] and multispecies [29] probiotic supplement) activates the T- and B-lymphocytes, increase cytokine (IFN-γ [20,25], IgA [29], IL-10 [27]) secretion, and suppressed the expression of pro-inflammatory cytokines (TNF- α, IL-6, IL-8) [27]. URTI is a common health issue among athletes. It has been proposed that immune cells can migrate from one site to another to provide protection, especially prevents the URTI [39]. The activation of Toll-like receptor (TLR)-2 by probiotics may activate the NF-κB pathway and innate immune signaling via MyD88, which provides better immune regulation and improved inflammatory cascades in elite athletes [40,41]. Further, a multigenus probiotic (*B. bifidum* W23, *B. lactis* W51, *E. faecium* W54, *L. acidophilus* W22, *L. brevis* W63, and *L. lactis* W58) mediated TLR-2 activation and enhanced the production of zonulin (tight junction protein), which may improve intestinal integrity [34].

**Figure 1:**
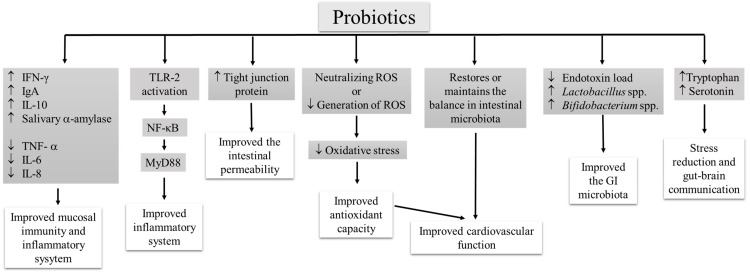
The possible mechanisms underlying the health-promoting properties of probiotics in athletes.

Strenuous exercise leads to cardiovascular modifications. Slight diastolic modifications and minor cardiac changes were observed in half-marathon runners [42]. Strenuous exercise induces cardiovascular sympathetic modulation in triathletes [43]. Beneficial effects of probiotics also include improvement of antioxidant levels to reduce the oxidative stress by decreasing the generation of ROS or neutralizing the ROS. Vasquez et al. [15] proposed that habitual intake of probiotics restores or maintains the balance in intestinal microbiota, reduces the oxidative stress, and exhibits beneficial cardiovascular effects. The improvement of radical scavenging potential was observed in athletes upon multispecies probiotic (*L. rhamnosus* IMC 501^®^ and *L. paracasei* IMC 502^®^) supplementation [28,29]. Improvement of gut microbiota by multispecies probiotic (*L. rhamnosus* IMC 501^®^ and *L. paracasei* IMC 502^®^) supplementation is one of the key mechanisms behind the health improvement in athletes [29]. Probiotic mediated improvement of intestinal permeability is one of the key mechanism in reducing the GI symptoms and reducing endotoxin content in athletes supplemented with multigenus probiotics (*L. acidophilus* CUL-60, *L. acidophilus* CUL-21, *B. bifidum* CUL-20, *B. animalis* subsp. *Lactis* CUL-34) along with prebiotic, and antioxidants [33]. According to Strasser et al. [35], probiotic (*B. bifidum* W23, *B. lactis* W51, *E. faecium* W54, *L. acidophilus* W22, *L. brevis* W63, and *L. lactis* W58) supplementation reduces the exercise-induced defect in tryptophan, and aids to increase the availability of serotonin, which improves the mental status of athletes and could establish gut–brain communication. The possible mechanisms behind the favorable effect of probiotic supplementation on athletes’ health have been summarized (Figure 1).

## 5. Factors Influencing the Effect of Probiotic Supplementation

However, several external factors influence the outcomes of the probiotic intervention. Dose, and duration of the intervention, formulation of probiotics and the ability of the strains are the highly influential factors.

The supplementation of an adequate amount of probiotics for a long duration confers health benefits. Nevertheless, the amount of probiotics for an athlete is the question, with several unclear answers. In fact, the required amount of probiotic is unique to every individual, and it is based on the physical, metabolic and mental status of the host.

Studies proved that a minimum intervention time is needed to prompt the positive immune response in athletes [21,22,23,24,44]. The multistrain probiotic formulation could increase the chance of adhesion and colonization of probiotic strain in the host GI tract. If the strains are compatible with each other, they confer synergetic effects. The use of multistrain probiotic formulations could reduce the duration of the supplementation. About four weeks supplementation of probiotic mix significantly improved the health status of rugby union players and runners in terms of incidence of URTI, GI discomforts, and inflammatory markers [32,45]. Cox et al. [25] reported that the supplementation of *L. fermentum* VRI-003 (1.2 × 10^10^ CFU per day for 4 weeks) reduced the severity and incidence of URTI and increased the whole blood culture’s IFN-γ concentration in elite distance runners. Health status of elite distance runners improved by the beneficial effect of probiotic supplementation, which is due to the ability of *L. fermentum* VRI-003 to colonize the intestinal tract.

The short time supplementation of probiotic did not enhance the athlete’s health. The administration of the high dose of probiotic (10^11^ cells) for short period (7 days) did not significantly improves the systemic cytokine profile and intestinal permeability when compared to the placebo group [46]. The studies revealed that the ability, combination, dose of probiotics and duration of intervention effectively influence the beneficial effects of probiotic supplementation in athletes.

## 6. Conclusions

Probiotic supplementation is recommended for athletes to maintain or to improve the health status and to overcome illness. The health-promoting property of the probiotic is specific to the species, strain and host. Some of the strains or combinations exhibited protective activity against GI symptoms and URTI. Most of the studies were conducted on *Lactobacillus* species like *L. rhamnosus, L. acidophilus, L. casei, L. helveticus, L. fermentum, L. plantarum,* and *L. salivarius*, but the results were not constant, and were dependent on the species, strain, dosage, duration and form of the interventions. Further studies are required to explore the beneficial effect of non-common probiotic strains in the sports context. The multispecies probiotic supplement (*L. rhamnosus* IMC 501^®^ and *L. paracasei* IMC 502^®^) improved intestinal microbiota [29], and the multigenus probiotic supplement (*L. acidophilus* CUL-60, *L. acidophilus* CUL-21, *B. bifidum* CUL-20, *B. animalis* subsp. *Lactis* CUL-34 [33]; Ecologic^®^ Performance containing *B. bifidum* W23, *B. lactis* W51, *E. faecium* W54, *L. acidophilus* W22, *L. brevis* W63, and *L. lactis* W58 [34]) improved intestinal permeability in athletes. 

Further studies are required to investigate the detailed mechanism behind the beneficial effects of probiotic intervention in athletes. Enhancement of mucosal immunity, improvement of intestinal permeability, positive regulation of gut microbial population, and production of secondary metabolites like short-chain fatty acids, are considered as the favorable contributions of probiotic supplements to the host system. Additional studies are also desired on the formulation and optimization of probiotic supplements to develop generalized and personalized sports supplements to boost the overall health and wellness of elite athletes.

## Figures and Tables

**Table 1:** Influence of probiotic supplementation on the health status of the athlete.

Subjects	Probiotics	Dose and Duration	Benefits/Impacts	References
Healthy marathon runners; n = 141 (16 females, 125 males); Age = 22 to 69 years	*Lactobacillus rhamnosus* GG	4 × 10^10^ CFU or 10^10^ CFU per day; 3 months	No changes in the incidence of GI-symptoms episodes, URTIs, and hematological parameters ↓ Duration of GI-symptoms episodes	Kekkonen et al. [19]
Healthy and fatigued athletes; n = 27 (10 females, 17 males); Age = 16 to 41 years	*L. acidophilus* LAFTI^®^L10	2 × 10^10^ CFU per day; 4 weeks	↑ Salivary IFN-γ ↑ IFN-γ secretion from WBC	Clancy et al. [20]
Endurance athletes; n = 84 (30 females, 54 males); Age = 18 to 55 years	*L. casei* Shirota	1.3 × 10^10^ CFU per day; 16 weeks	No change in severity of URTI ↓ Incidence of URTI ↑ Saliva IgA level	Gleeson et al. [21]
Endurance athletes; n = 268 (113 females, 155 males); Age = 18 to 32 years	*L. casei* Shirota	1.3 × 10^10^ CFU per day; 20 weeks	No influence on URTI, but improved the immune status	Gleeson et al. [22]
Elite athletes; n = 30 (6 females, 24 males); Age = 18 to 28 years	*L. helveticus* LaftiL10	2 × 10^10^ CFU per day; 14 weeks	Improved the immunity of athletes	Michalickova et al. [23]
Competitive cyclists; n = 62 (35 females, 64 males); Age = 25 to 45 years	*L. fermentum* VRI-003 (PCC^®^)	1 × 10^9^ CFU per day; 11 weeks	↑ *Lactobacillus* spp. count ↓ Severity of GI-symptoms in male ↓ Symptoms and illness load of URTIs in male No significant change in mucosal immunity	West et al. [24]
Elite distance runners; n = 20 males; Age = 20 to 35 years	*L. fermentum* VRI-003	1.2 × 10^10^ CFU per day; 28 days (1st treatment month)	↓ Severity and incidence of URTI ↑ Whole blood culture IFN-γ	Cox et al. [25]
Athletes; n = 51 males; Age = 20 years	*Lactococcus lactis* JCM 5805	10 × 10^10^ CFU per day; 13 days	↑ pDC maturation ↓ Incidence of URTI ↓ Fatigue	Komano et al. [26]
Triathletes; n = 18 (study 1); n= 16 (study 2); Age = 19 to 26 years	*L. plantarum* PS128	3 × 10^10^ CFU per day; 3 or 8 weeks	↓ Oxidative stress ↓ Pro-inflammation ↑ Anti-inflammation ↑ Plasma-branched amino acids	Huang et al. [27]
Amateur athletes; n = 24 males; Age = 25 to 39 years	*L. rhamnosus* IMC 501^®^, and *L. paracasei* IMC 502^®^ (1:1 ratio)	~10^9^ cells per day; 4 weeks	Neutralize the ROS ↑ Antioxidant potential	Martarelli et al. [28]
Athletes; n = 10 (7 females, 3 males); Age = 20 to 45 years	* *L. rhamnosus* IMC 501^®^ and *L. paracasei* IMC 502^®^	10^9^ CFU per strain; 2 × 10^9^ CFU per day; 4 weeks	Improve gut health, oxidative status, and mucosal immunity	Coman et al. [29]
Elite rugby union athletes; n = 19; Age = 20 to 35 years	*L. rhamnosus, L. casei, L. acidophilus, L. plantarum, L. fermentum, Bifidobacterium lactis, B. bifidum, Streptococcus thermophilus, Saccharomyces boulardi*	12 × 10^10^ CFU per day; 27 weeks	↓ Incidence of GI and URTIs ↑ Salivary α-amylase	Pumpa et al. [30]
Female endurance swimmers; n = 46; Age = 11 to 17 years	*L. acidophilus, L.* *delbrueckii* subsp. *bulgaricus, B.* *bifidum*, and *Streptococcus salivarius* subsp. *thermophilus*	400 mL per day (4 × 10^10^ per ml); 8 weeks	↓ Number of episodes of URTIs ↓ Dyspnea and ear pain	Salarkia et al. [31]
Rugby players; n = 30 males; Age = 19 to 29 years	*L. gasseri, B. bifidum, B. longum*	3 × 10^9^ CFU per day; 4 weeks	↓ Incidence of URTI ↓ GI episodes	Haywood et al. [32]
Long-distance triathletes; n = 30 (5 females, 25 males); Age = 30 to 38 years	** *L. acidophilus* CUL-60, *L. acidophilus* CUL-21, *B. bifidum* CUL-20, *B. animalis* subsp. *lactis* CUL-34	Total 30 × 10^10^ CFU per day; 12 weeks	↓ Endotoxin units ↓ GI symptoms Maintenance of intestinal permeability	Roberts et al. [33]
Trained athletes (triathletes, runners, cyclists); n = 23 males; Age = 30 to 45 years	*B. bifidum* W23, *B.* *lactis* W51, *Enterococcus faecium* W54, *L.* *acidophilus* W22, *L. brevis* W63, and *L. lactis* W58.	10^10^ CFU per day; 14 weeks	Improves intestinal permeability ↓ Exercise-induced protein oxidation ↓ TNF-α level	Lamprecht et al. [34]
Trained athletes; n = 29 (16 females, 13 males); Age = 20 to 35 years	*B. bifidum* W23, *B. lactis* W51, *E. faecium* W54, *L. acidophilus* W22, *L. brevis* W63, *L. lactis* W58	10^10^ CFU per day; 12 weeks	↓ Incidence of URTI ↓ Rate of exercise-induced tryptophan degradation	Strasser et al. [35]
Endurance athletes; n = 66 (38 female, 28 male); Age = 19 to 29 years	*L. salivarius*	2 × 10^10^ CFU per day; 4 months	No influence on URTI, and mucosal immunity	Gleeson et al. [36]
Healthy marathon runners; n = 139 (16 females, 123 males); Age = 30 to 50 years	*L. rhamnosus* GG	4 × 10^10^ CFU or 10^10^ CFU per day; 3 months	No significant change in the incidence of allergic diseases	Moreira et al. [37]

* Supplementation of probiotic along with prebiotic (oat bran fiber); ** supplementation of probiotic along with prebiotic (fructooligosaccharides) and antioxidant (α-lipoic acid and N-acetyl-carnitine); IFN-γ: Interferon-γ; WBC: White blood cells; CFU: Colony-forming units; GI: Gastrointestinal, ROS: Reactive oxygen species; URTI: Upper respiratory tract infection; pDC: Plasmacytoid dendritic cells.
